# Poly(methyl vinyl ether-*alt*-maleic anhydride) and Its Derivatives: From Polymer Synthesis to Advanced Biomedical Applications

**DOI:** 10.3390/polym18131667

**Published:** 2026-07-06

**Authors:** Pedro Valentín Badía-Hernández, Rocío Díaz-Puertas, Paula del Carmen Sánchez-García, Alberto Falcó, Pilar García-Morales, Ricardo Mallavia

**Affiliations:** 1 Institute for Research in Biotechnology and Health, Miguel Hernández University, Avda de la Universidad s/n, 03202 Elche, Spain; pbadia@umh.es (P.V.B.-H.); r.diaz@umh.es (R.D.-P.); paula.sanchez15@goumh.umh.es (P.d.C.S.-G.); pgarcia@umh.es (P.G.-M.); 2 Fish Pathology Group, Institute of Aquaculture Torre de la Sal—Spanish National Research Council (IATS-CSIC), Ribera de Cabanes, 12595 Castellon, Spain; alberto.falco@csic.es

**Keywords:** PMVEMA, crosslinking, functionalization, biocompatibility, biodegradability, bioadhesion, drug delivery

## Abstract

Poly(methyl vinyl ether-*alt*-maleic anhydride) (PMVEMA) is a versatile synthetic copolymer that has gained considerable attention in biomedical and pharmaceutical applications due to its biocompatibility, biodegradability, bioadhesive properties and chemical reactivity. This review summarizes the current knowledge regarding the derivatives, physicochemical properties, functionalization and crosslinking strategies of PMVEMA, with particular emphasis on their relevance to biomedical applications. A comprehensive literature analysis was performed using major scientific databases, combined with artificial intelligence-assisted text mining, to identify the principal research trends associated with PMVEMA. The reviewed studies demonstrate that the reactive anhydride groups of PMVEMA enable the formation of a wide variety of derivatives, including hydrogels, nanoparticles and nanofibers with tunable properties. These characteristics have facilitated its application in different fields, including immunology, drug delivery, dentistry and dermatology. In particular, PMVEMA-based systems exhibit enhanced mucosal adhesion, controlled drug release, immunoadjuvant activity and biocompatibility in vitro and in vivo. Despite its broad applicability, further studies are still needed to fully elucidate its biodegradation mechanisms in vivo and optimize its clinical translation.

## 1. Introduction

Biodegradable and biocompatible polymers have attracted considerable attention in biomedical research due to their ability to degrade into non-toxic or easily metabolized products while maintaining compatibility with biological systems, thereby minimizing adverse effects and long-term accumulation in the body [1,2]. Among these materials, poly(methyl vinyl ether-*alt*-maleic anhydride) (PMVEMA, also referred to as PVM/MA) is widely recognized as a versatile synthetic polymer with a broad range of pharmaceutical and biomedical applications.

PMVEMA (CAS No. 9011-16-9) is an alternating copolymer composed of methyl vinyl ether and maleic anhydride repeating units. Its highly regular structure arises from the strong electron-accepting character of maleic anhydride, which promotes alternating free-radical copolymerization with electron-donating vinyl ethers according to the charge-transfer model [3]. Typically, PMVEMA is synthesized via free-radical polymerization under relatively mild conditions, employing initiators such as dibenzoyl peroxide or dilauroyl peroxide. The alternating structure in chain copolymerization results from the combined effects of both the monomer polarity differences and the relative stability of the propagating radical species formed, particularly the electron-accepting character of maleic anhydride compared with other vinyl monomers [4].

**Figure 1:**
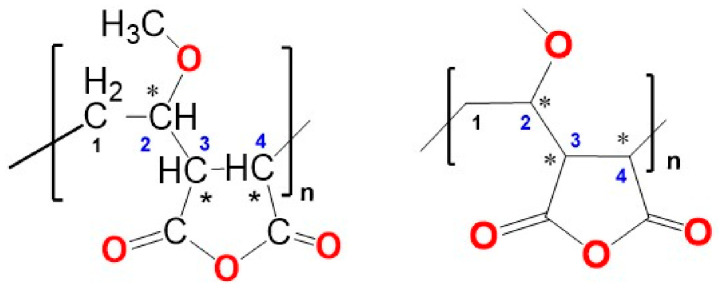
Chemical structure of the PMVEMA repeating unit, shown as expanded and semi-expanded formulas. Asterisks indicate asymmetric carbons.

Furthermore, the resulting structural unit features three chiral centers along the same main chain (C2, C3, and C4), which may influence its final properties (Figure 1). From a characterization standpoint, up to eight possible stereoisomers with defined absolute spatial configurations may be present, in addition to configurational sequences differing in tacticity at C2, including isotactic, syndiotactic, and, most frequently, atactic polymers. These features complicate the structural elucidation of this unit by nuclear magnetic resonance (NMR) spectroscopy.

Polymerization can be carried out in various organic solvents, including benzene and toluene, yielding high-molecular-weight polymers, whereas solvents such as acetone generally lead to lower-molecular-weight products with reduced viscosity [5]. PMVEMA is commercially available under trade names such as Gantrez^®^, produced by Ashland Inc. (Wilmington, DE, USA). Depending on its molecular weight and degree of hydrolysis, the polymer can be obtained in different chemical forms, including the anhydride form (PMVEMA), the fully or partially hydrolyzed acid form, poly(methyl vinyl ether-*alt*-maleic acid) (PMVEMA-Ac), and various ester derivatives, like poly(methyl vinyl ether-*alt*-monoethyl-ester) (PMVEMA-Es).

From a regulatory and safety perspective, the PMVEMA polymer and related derivatives have documented uses in products intended for human use. In the United States, these uses are associated with specific regulatory provisions and conditions of use. For example, PMVEMA is listed in the U.S. Food and Drug Administration (FDA) Inventory of Food Contact Substances Listed in Title 21 of the Code of Federal Regulations (CFR) under 21 CFR 175.105 for use in adhesive applications involving food-contact articles. In addition, certain PMVEMA-Ac copolymer-containing denture adhesive formulations are regulated as dental prosthetic devices under 21 CFR 872.3500 [6]. Likewise, PMVEMA and certain derivatives are listed as cosmetic ingredients in the European Commission’s CosIng (Cosmetic Ingredients) database, with reported functions including antistatic, binding, emulsion-stabilizing, film-forming, hair-fixing, and viscosity-controlling properties [6,7,8].

However, despite the current use of PMVEMA, it was placed in 2025 on the official list of the Federal Food, Drug, and Cosmetic Act (FD&C Act), known as the 503B Bulks List, under “Category 3: Bulk Drug Substances Nominated Without Adequate Support;” therefore, its use in compounded drug formulations is not permitted [9]. Nevertheless, both the Cosmetic Ingredient Review (CIR) Expert Panel and the European Food Safety Authority (EFSA) have issued favorable safety assessments for these materials under defined conditions of use. The CIR Expert Panel concluded that PMVEMA and its related salts and esters are safe for use in cosmetic products, whereas the EFSA concluded that PMVEMA-Ac is safe as a novel food ingredient under certain uses and use levels [6,7,10].

Compared with other polymers, PMVEMA is distinguished by its unique combination of properties. Unlike widely used biocompatible polymers such as poly(ethylene glycol) (PEG), poly(vinylpyrrolidone) (PVP), and poly(lactic-co-glycolic acid) (PLGA), which are often employed as inert carriers or structural matrices, PMVEMA contains abundant reactive sites that facilitate chemical conjugation, while also displaying intrinsic mucoadhesive behavior, antimicrobial activity and biocompatibility [11]. These features have made PMVEMA a particularly attractive platform for the development of advanced drug delivery systems or immunological applications [12]. Accordingly, the incorporation of additional materials or functional moieties into PMVEMA-based systems represents an important strategy for tuning and enhancing their physicochemical and biological properties, particularly in biomedical applications where the native properties of PMVEMA require further modulation to overcome specific limitations.

As a result of these properties, PMVEMA has been extensively explored in a variety of biomedical applications, including drug delivery systems, mucoadhesive formulations, dental materials, wound healing platforms and controlled release technologies. Its reactive anhydride groups further enable the synthesis of polymer-drug conjugates and functionalized systems tailored to specific therapeutic needs.

Although PMVEMA has been well described and extensively characterized for decades, its use in biomedical applications has expanded substantially in recent years. The objective of this review is to summarize and critically discuss the physicochemical properties of PMVEMA described in bibliography, with particular emphasis on those that influence its main applications. Furthermore, recent advances in the biomedical applications of this polymer are discussed, highlighting its current limitations as well as its potential for further development across various fields.

## 2. Materials and Methods

A comprehensive bibliographic search was conducted to identify relevant publications on PMVEMA. The search was performed using major scientific databases, including Scopus, MEDLINE, and Web of Science, on 5 March 2026. The following keywords and their combinations were used: “PMVEMA” OR “Gantrez” OR “poly(methyl vinyl ether-*alt*-maleic anhydride)” AND biomedical. No restrictions on publication date were initially applied to capture the full scope of the available literature.

A total of 411 articles were retrieved from the selected databases. After removal of duplicates, the resulting dataset was analyzed using a natural language processing (NLP)-based artificial intelligence (AI) tool developed by Álvarez-Martínez et al. (2023), implemented as a Google Colab notebook available with the publication [13]. This tool enabled automated text mining and clustering of bibliographic information based on semantic similarities.

**Figure 2:**
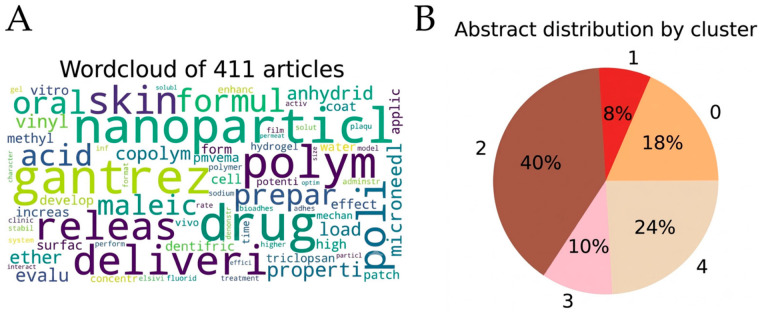
(**A**) Word cloud illustrating the most frequently occurring terms in the articles included in this review. (**B**) Distribution of articles across the identified clusters.

The analysis generated the word cloud representing the most frequently occurring terms across the retrieved manuscripts (Figure 2A), providing a visual overview of the dominant research themes. Five distinct clusters were identified, each characterized by the following predominant terms: (i) “nanoparticles and oral delivery” (Cluster 0), (ii) “vaccines and immunology” (Cluster 1), (iii) “polymeric drug delivery systems” (Cluster 2), (iv) “dentifrice and triclosan” (Cluster 3), and (v) “skin applications and microneedling” (Cluster 4). The distribution of articles across the different clusters is shown in Figure 2B. Cluster 0 was systematically used throughout the article, whereas clusters 1–4 were employed for the specific biomedical applications discussed in Section 6.

**Figure 3:**
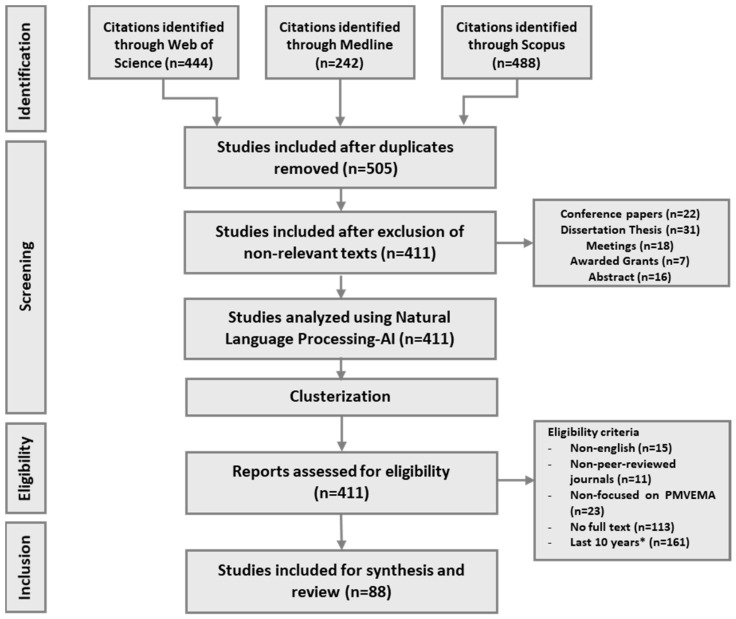
Flow diagram illustrating the literature search, screening, eligibility assessment, and study selection process used in this review. * When multiple studies addressing the same topic were available, preference was given to the most recent publications. This criterion was not applied to research areas with limited available literature, where older studies were also included to ensure comprehensive coverage of the field.

Articles within each cluster were subsequently screened for relevance. The inclusion criteria were as follows: (i) publications in English; (ii) articles published in peer-reviewed journals; (iii) studies focused on PMVEMA chemistry, synthesis, properties or applications; (iv) preferential availability in open-access format; (v) for articles within the same field, priority was given to the most recent publications and to studies addressing different topics in order to represent a broader range of research. Studies were selected based on their relevance to the topic and their compliance with these predefined criteria. The complete literature selection process is summarized in the flow diagram shown in Figure 3.

## 3. Functionalization and Crosslinking Strategies

### 3.1. PMVEMA Derivatives

**Figure 4:**
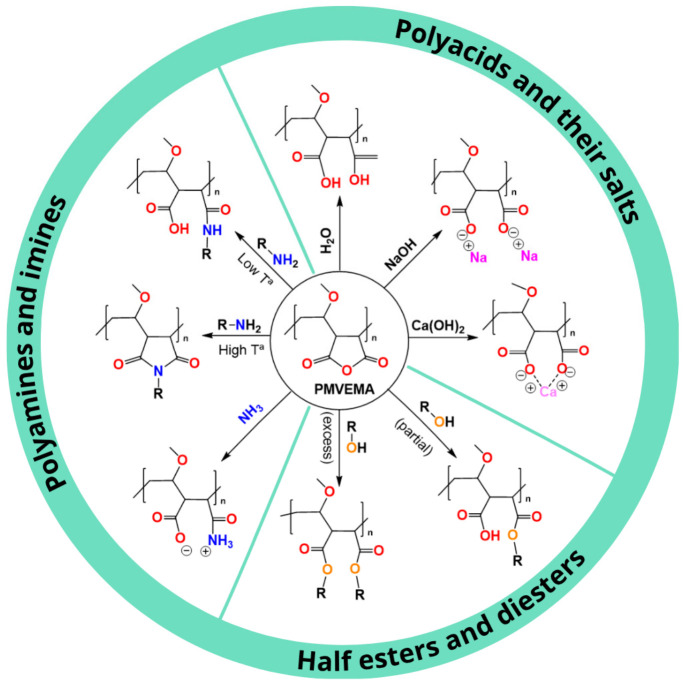
Schematic representation of PMVEMA reactivity according to the type of derivative obtained.

PMVEMA exhibits a high degree of chemical reactivity due to the presence of its anhydride functional groups, which readily undergo reactions with a wide range of nucleophilic species, including water, alcohols, epoxides, ammonia, and primary or secondary amines. These reactions enable the formation of a diverse array of structurally and functionally versatile derivatives (Figure 4).

Upon dissolution in aqueous media, the anhydride rings undergo hydrolysis to yield the corresponding dicarboxylic acid groups. In aqueous salt solutions, PMVEMA can form polyacid salt derivatives, particularly in the presence of alkali metal hydroxides (NaOH) or alkaline earth metal hydroxides (Ca(OH)_2_). In the case of divalent cations, complexation between the metal centers and the maleic acid moieties may occur in addition to electrostatic interactions.

The formation of monoester and diester derivatives is consistent with the well-established nucleophilic ring-opening reactivity of maleic anhydride copolymers toward alcohols, where partial alcoholysis yields half-esters, while further esterification under excess alcohol or catalytic conditions leads to fully esterified diester structures.

Similarly, in the presence of ammonia or primary and secondary amines, the anhydride linkage undergoes nucleophilic ring opening, leading to the formation of ammonium salts, half-amide salts, or poly(amic acid) intermediates. Depending on the reaction conditions and the nature of the nucleophile, these intermediates may further undergo cyclodehydration to yield polyimide structures. The relative proportion of amic acid and imide units within the modified polymer can be effectively controlled by the reaction temperature. Lower temperatures favor the formation of amic acid functionalities, whereas higher temperatures promote cyclization and dehydration, resulting in an increased content of imide units. These reactions allow covalent conjugation with biomolecules such as lectins, as well as with therapeutic agents such as doxorubicin [14].

In addition to these reactions, PMVEMA can interact with nonionic surfactants, leading to the formation of internally plasticized materials. These systems are characterized by their ability to produce flexible, transparent films with relatively low tensile strength. Furthermore, PMVEMA reacts with specific functional compounds, such as chalcones and hydantoins, to yield resinous ester derivatives. These materials are of particular interest due to their photosensitivity, which makes them suitable for applications in photomechanical reproduction processes.

### 3.2. Crosslinking

**Figure 5:**
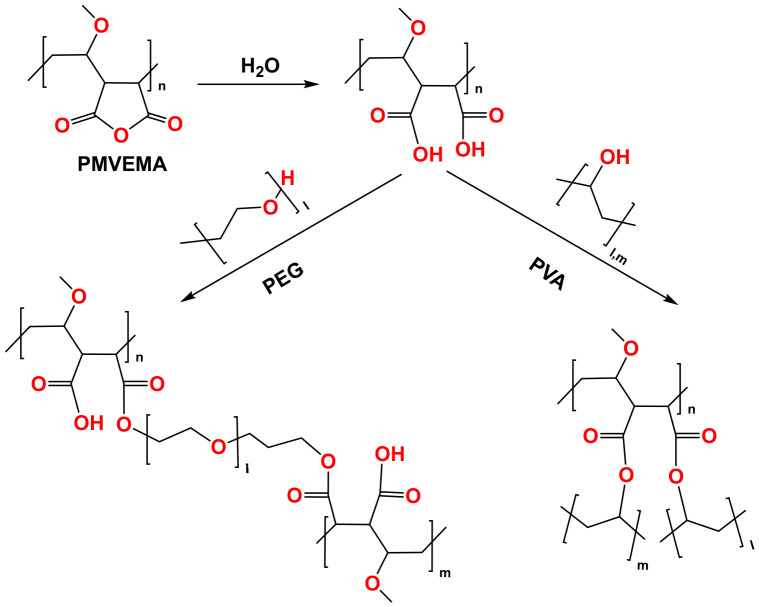
PMVEMA crosslinking reactions with PEG and PVA.

PMVEMA exhibits significant crosslinking capability owing to the high reactivity of its cyclic anhydride groups toward nucleophilic and multifunctional reagents [15]. Covalent crosslinking typically proceeds through reactions with bifunctional or polyfunctional compounds, such as polyamines and polyhydroxy compounds, leading to the formation of three-dimensional network structures with enhanced mechanical strength and thermal stability [16]. For instance, diamines, such as ethylenediamine or hexamethylenediamine, react with anhydride moieties to form amide linkages and poly(amic acid) intermediates, which may further cyclize into polyimide networks upon thermal treatment [17,18]. Similarly, polyols, such as glycerol, PEG or poly vinyl alcohol (PVA), promote crosslinking via esterification reactions, yielding polyester-type networks [19]. The crosslinking reaction of PMVEMA with PEG and PVA is shown in Figure 5. Additional crosslinking can be achieved using a wide range of multifunctional vinyl or acrylic compounds, such as divinyl ethers of aliphatic diols [20,21], divinylbenzene [22], polyethylene glycol diacrylate [23] or N,N′-methylenebisacrylamide [24], which enable network formation through radical or addition mechanisms. Epoxy compounds also serve as effective crosslinkers by reacting with carboxylic acid groups generated after partial hydrolysis of the anhydride units [25].

Beyond covalent crosslinking, PMVEMA has the capacity to form physical or coordination-based networks. In its hydrolyzed form, the carboxylate groups impart a high anionic charge to PMVEMA, enabling it to coordinate with metal cations, with a particularly high affinity for divalent and trivalent metals (e.g., Ca^2+^, Mg^2+^, Mn^2+^, Zn^2+^, Fe^3+^) [26,27,28].

In addition, PMVEMA forms intermolecular complexes with natural biomacromolecules, such as cellulose [29], pectin [30], or chitosan [31], giving rise to highly viscous, gel-like systems. These interactions, which may involve hydrogen bonding and charge transfer, are particularly relevant in cosmetic and pharmaceutical formulations. The resulting crosslinked or complex materials are often water-insoluble and find applications such as protective coatings, binding agents, and permanent finishes. In some cases, post-crosslinking hydrolysis and neutralization of residual anhydride groups are performed to tailor hydrophilicity, swelling behavior and biocompatibility [32].

## 4. Properties of PMVEMA

The widespread use of PMVEMA in pharmaceutical and biomedical formulations is closely associated with its favorable combination of physicochemical and biological properties, which are discussed in this section and summarized in Table 1. Potential limitations affecting the use of PMVEMA in biomedical applications are also addressed.

### 4.1. Bioadhesion

PMVEMA exhibits pronounced bioadhesive properties that are largely attributed to the hydrolysis of its anhydride bonds into carboxylic acid groups, which can form hydrogen bonds with mucosal components, such as mucins. Irache et al. (2005) demonstrated that this adhesive capacity is enhanced when the polymer is formulated as nanoparticles rather than in its soluble form [34]. Following oral administration, PMVEMA nanoparticles show a clear tropism for the upper gastrointestinal tract, particularly the stomach and jejunum. Furthermore, the bioadhesive behavior of PMVEMA can be modulated through chemical modifications and surface functionalization. For instance, cross-linking with 1,3-diaminopropane increases nanoparticle stability but reduces adhesion by blocking the carboxylic groups responsible for mucosal interactions [34]. Surface coatings also influence targeting: while gelatin coatings tend to decrease overall interaction with the mucosa, bovine serum albumin (BSA) coatings promote a stronger affinity for the gastric mucosa [34]. Additionally, these interactions are dose-dependent: lower doses favor a higher proportion of adhesion, whereas higher doses may saturate available binding sites in the gastrointestinal tract, thereby reducing relative attachment efficiency.

### 4.2. Biocompatibility

PMVEMA is known to be biocompatible and therefore suitable for pharmaceutical and food applications. Some evaluations of PMVEMA nanoparticles demonstrate that they generally do not compromise cell metabolism, membrane integrity or viability in various cell lines, such as human Caco-2 from colorectal adenocarcinoma and HT29-MTX, a subclone from human colorectal adenocarcinoma cell line HT29, selected through methotrexate, even at concentrations as high as 2 mg/mL [39]. Furthermore, research indicates that the polymer lacks significant genotoxic or mutagenic potential, as it does not induce biologically relevant DNA strand breaks or point mutations in mouse lymphoma cells (at concentrations up to 600 μg/mL), nor does it show mutagenicity in Ames tests [33]. Scientific safety opinions concluded that PMVEMA is safe for human consumption as a chewing gum base ingredient, noting a high no-observed-adverse-effect level (NOAEL) of approximately 1.8 to 2.1 g/kg of body weight per day in subchronic rat studies [10]. Biocompatibility is further supported by evidence that the polymer is not systemically absorbed to any significant degree, remains localized in the gastrointestinal tract, and exhibits cytoadhesion to cell surfaces without internalization [33]. While one in vivo study observed localized DNA damage in the duodenal tissue of mice at an extreme limit dose of 2000 mg/kg of body weight, no such effects were seen at lower therapeutic doses, and histopathological analysis confirmed that the tissue remained healthy and undamaged [33].

### 4.3. Degradability

Although the degradability of PMVEMA is frequently cited as a major advantage in pharmaceutical formulations, the literature specifically describing its degradation mechanism under physiological conditions remains limited.

The aqueous degradation behavior of PMVEMA is initially governed by the hydrolysis of its highly reactive maleic anhydride groups. Upon exposure to aqueous or biological media, water molecules can nucleophilically attack the anhydride moieties (–CO–O–CO–), leading to ring opening and the formation of dicarboxylic acid groups [36]. However, this hydrolytic conversion should be distinguished from degradation of the polymer backbone. Studies investigating the stability of PMVEMA in aqueous media have shown that a decrease in molecular weight can occur over time, particularly at elevated temperatures and mildly acidic conditions (pH < 7), suggesting the occurrence of chain scission processes [36]. Ladavière et al. (1999) [36] further reported the formation of degradation products, including CO_2_ and methanol, during thermal degradation in solution, together with spectroscopic changes consistent with structural rearrangements of the polymer. Polymer chain mobility may also influence the degradation process. More flexible chains, typically associated with lower molecular weight PMVEMA, may facilitate the conformational rearrangements involved in hydrolysis and subsequent degradation steps, leading to faster degradation rates than those observed for higher molecular weight polymers.

This chemical hydrolysis contributes to the subsequent bioerosion of the formulation, a physical process involving mass loss and dissolution [40]. In polyanhydride-based systems, hydrolytic cleavage of labile anhydride groups can occur faster than water diffusion into the polymer matrix, thereby favoring surface erosion in macroscopic systems such as films or disks [40]. Under these conditions, degradation and mass loss are mainly confined to the material–fluid interface, which may enable relatively predictable and controlled release kinetics [40]. However, the bioerosion profile is strongly influenced by the geometry, composition, and hydration behavior of the system. In nanoparticulate PMVEMA-based systems, the high surface-to-volume ratio facilitates rapid water penetration, anhydride hydrolysis, and polymer dissolution, resulting in much faster bioerosion than that observed in larger matrices [41]. Moreover, additives such as encapsulated drugs can considerably affect both the rate of hydrolysis and the subsequent bioerosion process [42].

When PMVEMA is used as a cross-linking agent with other polymers, such as PEG or nanocellulose, the opening of the anhydride rings can generate ester linkages with hydroxyl-containing structures [43]. Although these cross-linked materials exhibit improved stability in aqueous environments, their long-term degradation may still proceed through the hydrolytic cleavage of the newly formed ester bonds, together with the hydrolysis of any residual anhydride groups.

The ultimate physiological fate of PMVEMA remains insufficiently characterized, and only limited information is available regarding the long-term disposition of the polymer and its degradation products in vivo. Based on its chemical structure and the hydrolytic degradation mechanisms described above, PMVEMA is unlikely to undergo extensive enzymatic metabolism. Instead, hydrolysis and bioerosion are expected to progressively reduce the polymer into lower-molecular-weight, highly water-soluble carboxylated fragments, which may subsequently be eliminated through normal physiological clearance pathways, such as renal excretion. However, direct experimental evidence supporting this proposed clearance mechanism remains scarce. Further studies are therefore needed to elucidate the metabolic fate, biodistribution, elimination pathways, and potential accumulation of PMVEMA degradation products in biological systems and the environment.

### 4.4. Antimicrobial

Several studies suggest that PMVEMA may also exhibit intrinsic antimicrobial properties. Unlike conventional antimicrobial materials, which rely on the incorporation of active agents, the antimicrobial effects of PMVEMA appear to be mainly associated with its physicochemical characteristics.

This activity has been largely attributed to the acidic nature of PMVEMA. Upon exposure to aqueous environments, hydrolysis of the maleic anhydride groups leads to ring opening and the formation of carboxylic acid groups, which can lower the local pH to values typically ranging from 3.0 to 5.2. In PMVEMA-containing cryogel and hydrogel formulations, this acidic environment has been associated with the inhibition of *Staphylococcus aureus* [37,44].

Beyond its acidic properties, PMVEMA may also act as an inhibitor of bacterial adhesion, a critical step in biofilm development. In dental and orthodontic contexts, the copolymer has been shown to markedly reduce the retention of *Streptococcus mutans* on surfaces such as enamel [38] and acrylic resins [45]. This anti-adhesive effect has been attributed to a dual mechanism involving divalent calcium ion (Ca^2+^) chelation and enhanced electrostatic repulsion. By chelating Ca^2+^ ions at the surface, PMVEMA may disrupt the salt bridges required for initial bacterial attachment to the acquired salivary pellicle. Furthermore, the polyanionic nature of the polymer can create repulsive interactions with the anionic bacterial membranes, with observed reductions of bacterial coverage of up to 93% under the tested conditions [38].

### 4.5. Limitations

Despite its numerous advantages, the performance of PMVEMA-based systems is often constrained by the polymer’s intrinsic chemical characteristics, which may limit their use in specific biomedical applications. Nevertheless, substantial research efforts have been devoted to overcoming these drawbacks through chemical modification and formulation engineering. The main limitations and the corresponding strategies proposed in the literature are summarized in Table 2.

One important limitation of PMVEMA is its relatively low capacity to load highly lipophilic drugs. The hydrophilic nature of the polymer often results in insufficient loading of poorly water-soluble compounds, such as glibenclamide [46,47], camptothecin [48] and rifampicin [49], thereby limiting the achievable therapeutic dose. To address this issue, several approaches have been explored, including the covalent grafting or co-encapsulation of cyclodextrins, particularly hydroxypropyl-β-cyclodextrin (HP-β-CD), which can accommodate hydrophobic molecules within their cavities and substantially increase drug loading [46,47]. Similarly, the preparation of polymer–drug conjugates based on methoxy poly(ethylene glycol) (mPEG) has been shown to enhance the incorporation efficiency of lipophilic drugs compared with unmodified PMVEMA-based carriers [48].

Another challenge arises from the strong bioadhesive properties of PMVEMA. Although mucoadhesion is advantageous for increasing residence time at mucosal surfaces, excessive adhesion may hinder nanoparticle diffusion through the mucus layer and limit access to the underlying epithelium [54]. This effect is particularly relevant for oral delivery systems intended to reach enterocytes or Peyer’s patches [49]. To overcome this limitation, surface-engineering strategies have been developed to generate mucus-permeating nanoparticles. PEGylation, either through surface coating or covalent attachment of PEG derivatives, reduces interactions with mucins and promotes diffusion through the mucus barrier [50]. Alternative approaches include coating nanoparticles with galactose-containing polysaccharides to promote intestinal transit [51] or incorporating hydrophobic excipients, such as ethyl cellulose, to reduce mucoadhesion and enhance localization within intestinal lymphoid tissues [49].

A further limitation of PMVEMA-based systems is their relatively rapid hydration and hydrolysis in aqueous environments, which can result in accelerated drug release and reduced formulation stability. In some cases, unmodified nanoparticles may undergo extensive erosion or dissolution within 24 h, compromising sustained-release performance [52]. Several stabilization strategies have therefore been investigated. Chemical crosslinking with agents such as 1,3-diaminopropane increases matrix stability by reducing the availability of hydrolyzable groups and restricting polymer-chain mobility, thereby prolonging residence time [52]. Likewise, ionic crosslinking with divalent cations, including magnesium and calcium ions, enhances nanoparticle stability and extends their persistence under physiological conditions [48,53]. Moreover, cyclodextrin-grafted PMVEMA conjugates can further modulate release kinetics by generating biphasic profiles, in which surface-associated drug is released rapidly, while drug molecules complexed within cyclodextrin cavities are released more gradually [47].

## 5. PMVEMA as a Matrix for Biomaterials

The versatility of PMVEMA has enabled its integration with various natural and synthetic polymers to develop advanced materials with applications ranging from regenerative medicine to water purification and optoelectronics.

### 5.1. Hydrogels

The structure of PMVEMA allows for the formation of stable three-dimensional networks through hydrogen bonding. This crosslinking ability, when controlled under specific synthesis conditions, enables the development of hydrogels in combination with other polymers or crosslinking agents.

Crosslinking can be achieved through thermal transesterification with hydroxyl-containing polymers such as hyaluronic acid (HA) [55], where heat promotes ester bond formation between carboxyl and hydroxyl groups. Chemical crosslinking strategies, particularly 1-ethyl-3-(3-dimethylaminopropyl)carbodiimide (EDC)/(N-hydroxysuccinimide) (NHS)-mediated reactions, further enhance network formation by activating PMVEMA carboxyl groups to react with primary amines present in polymers, such as gelatin [56].

These crosslinked hydrogel systems exhibit remarkable physicochemical and mechanical properties, including high structural stability, bioadhesion, viscoelasticity, flexibility and water retention capacity. For example, PMVEMA-based hydrogels with chitosan and HA maintain structural integrity under physiological conditions for extended periods and support fibroblast colonization in tissue engineering applications [55]. Similarly, Singh et al. (2010) synthesized PEG/PMVEMA hydrogels with porous morphology and strong water retention, showing PMVEMA-content-dependent changes in swelling behavior and solute permeation, highlighting their potential application in controlled drug delivery systems [57]. Moreover, EDC/NHS-crosslinked gelatin–PMVEMA hydrogels produced flexible and translucent bioadhesive matrices with high swelling capacity and suitable mechanical properties for topical drug delivery, including the transport of nanocarriers such as curcumin-loaded liposomes [56].

### 5.2. Nanoparticles

PMVEMA-based nanoparticles can be synthesized using several established techniques, including nanoprecipitation and emulsification–solvent evaporation. In nanoprecipitation, when using PMVEMA as the primary matrix, it is dissolved in an organic solvent, such as acetone, and is then added under agitation to a hydroalcoholic phase containing a crosslinker, for instance, 1,3-diaminopropane (DP) [58]. Emulsification methods have also been used to develop hybrid nanoparticles based on a modified PMVEMA. In one example, PMVEMA–D-α-Tocopheryl polyethylene glycol succinate (TPGS) was blended with PLGA and glyceryl monostearate to improve the mucin adhesion properties of the material [59].

Furthermore, PMVEMA serves as a versatile coating material and platform for nanoparticle surface functionalization. It has been used to stabilize albumin- [60] and zein-based nanoparticles [61], preserving the activity of encapsulated therapeutics and improving oral immunogenicity. PMVEMA can also be covalently functionalized with hydroxypropyl-β-cyclodextrin (HP-β-CD) via a Steglich-type reaction using EDC as the coupling agent. This conjugated polymer, when blended with PEG, enables the preparation of nanoparticles that significantly increase the drug-loading capacity for lipophilic drugs, such as glibenclamide, using the solvent displacement technique [46], or camptothecin, using the nanoprecipitation technique [48].

As discussed in the properties section, uncoated PMVEMA nanoparticles are strongly mucoadhesive; however, PEGylation or selected biopolymer coatings can generate a hydrophilic corona that promotes mucus permeation, allowing nanoparticles to diffuse through the mucus layer and reach the intestinal epithelium [62].

### 5.3. Nanofibers

Electrospinning is the most widely used method for producing PMVEMA-based nanofibers because it is simple, scalable, affordable and capable of generating nanofibers that mimic the extracellular matrix [63]. Under optimized conditions, PMVEMA formulations can produce uniform fibers with diameters below 1000 nm and high drug-loading capacities, achieving encapsulation efficiencies close to 100% for compounds such as 5-aminolevulinic acid (5-ALA) [64] or antibiotics such as amikacin, ciprofloxacin, cefotaxime or neomycin [65]. Drug release from these systems generally follows Higuchi kinetics and Fickian diffusion, making them suitable for sustained-release applications.

PMVEMA is frequently combined with other polymers to improve mechanical properties and release kinetics. For example, blending PMVEMA with PLGA enables controlled drug release while enhancing hydrophilicity and cell adhesion, making these fibers attractive for wound-dressing applications [66]. In addition to supporting cell adhesion and proliferation, PMVEMA nanofibers have been shown to exhibit intrinsic hemostatic activity by promoting platelet adhesion [66].

## 6. Biomedical Applications

The unique physicochemical properties of PMVEMA make it a versatile platform for biomedical applications. Owing to these characteristics, its use can be broadly categorized into four principal areas of application: dentistry, immunology, drug delivery and dermatology (Figure 2). Each of these fields exploits specific features of the polymer, such as its capacity for chemical modification, bioadhesion, or interaction with biological systems. A more detailed discussion of these application areas is provided in the following sections.

### 6.1. Immunology

PMVEMA has been widely explored in immunology and vaccine delivery applications. Its primary utility arises from the presence of highly reactive anhydride groups, which enable the straightforward conjugation of antigens, proteins (such as allergens), and targeting ligands without the need for complex chemical activation.

Beyond its role as a passive delivery vehicle, PMVEMA nanoparticles have been shown to act as active Th1-profile adjuvants. Tamayo et al. (2010) demonstrated that these nanoparticles function as agonists for Toll-like receptors (TLR2, TLR4 and TLR5), triggering the maturation of antigen-presenting cells (APCs) such as dendritic cells (DC) and macrophages [67]. This activation leads to a significant upregulation of co-stimulatory molecules (CD54 and CD86) and the secretion of pro-inflammatory cytokines, including interleukin-12 (IL-12) and tumor necrosis factor (TNF-α). Furthermore, PMVEMA nanoparticles showed the capacity to elicit CD8+ T-cell responses in vivo studies in mice. Further studies by Camacho et al. (2011) showed that PMVEMA nanoparticles also activated the complement system via surface hydroxyl groups on their surface, enabling covalent deposition of C3b and consumption of complement activity, which enhances opsonization and recruitment of antigen-presenting cells [68]. The combined activation of TLR signaling and complement pathways suggests a synergistic mechanism by which PMVEMA nanoparticles bridge innate and adaptive immunity, thereby functioning as potent intrinsic adjuvants. However, most of the available evidence has been generated in preclinical animal models, and caution should be exercised when extrapolating these findings to humans. The magnitude and quality of immune responses may depend strongly on formulation characteristics, administration route, and dose, whereas excessive activation of innate immune pathways could potentially lead to undesirable inflammatory or immunotoxic effects.

PMVEMA has also demonstrated strong potential for mucosal immunization, largely due to its intrinsic bioadhesive properties, which prolong antigen residence time at mucosal inductive sites and enhance immune activation. Its adjuvant effect has been validated across different administration routes. For instance, Gómez et al. (2007) showed that orally administered PMVEMA nanoparticles loaded with ovalbumin significantly enhanced immune responses and protected mice from anaphylactic shock [69]. In intranasal and orotransmucosal delivery, derivatives such as PMVEMA-Pluronic F127 conjugates can form thermosensitive, mucoadhesive hydrogels. These systems undergo a liquid-to-gel transition at body temperature, enhancing antigen residence in the nasal cavity and targeting the nasopharynx-associated lymphoid tissue (NALT) [70]. In intradermal delivery, blends of PMVEMA have been used to fabricate dissolving microneedles (MNs). These needle-free devices bypass the stratum corneum to deliver antigens directly to the skin-associated lymphoid tissue (SALT), providing a painless and self-administrable alternative to traditional injections [71].

The chemical versatility of PMVEMA allows for surface modification with various ligands to enhance targeting specificity. For example, mannosamine-coated nanoparticles target mannose receptors on dendritic cells, mimicking the entry mechanisms of certain pathogens [72]. Similarly, the association of flagellin with PMVEMA nanoparticles acts as both a mucoadhesin and a TLR5 agonist, further shifting the immune response toward a Th1 subset [73]. Other ligands, such as wheat germ agglutinin (WGA), have been utilized to improve bioadhesion to enterocytes [74].

### 6.2. Drug Delivery

Due to its distinctive properties, PMVEMA has been used as a polymeric platform for drug delivery. Specifically, its hydrolyzable anhydride groups enable strong interactions with biological membranes and mucosal surfaces, prolonging drug residence time and enhancing absorption, particularly in mucosal administration routes. The literature has shown that PMVEMA biomaterials can encapsulate a wide range of therapeutic agents, including proteins, peptides, vaccines, and small molecules, while protecting them from degradation. These studies are summarized in Table 3.

The most significant application of PMVEMA in the literature is the enhancement of oral bioavailability for drugs with poor solubility or high first-pass metabolism. For example, a study by D’Souza et al. (2016) showed that curcumin-loaded PMVEMA nanoparticles exhibited a 4- to 5-fold increase in gastroretention and a 10-fold relative enhancement in oral bioavailability compared with standard suspensions, attributed to bioadhesion [51]. PMVEMA-based systems often target Peyer’s Patches in the gut-associated lymphoid tissue, allowing intact nanoparticles to enter the lymphatic system and bypass hepatic degradation [49]. This strategy resulted in an 18-fold increase in the oral bioavailability of berberine [75] and enhanced intestinal absorption of insulin [76] and cabazitaxel [59].

PMVEMA is also extensively used to create biomaterials for localized therapy. A study by Badía-Hernández et al. (2025) showed that electrospun PMVEMA nanofibers designed for local, post-surgical treatment of glioblastoma, provided sustained release of antineoplastic drugs, such as carmustine (BCNU) and doxorubicin (DOX), while slowing their degradation under physiological conditions [77]. Similarly, 5-aminolevulinic acid (5-ALA) encapsulated in PMVEMA nanofibers remained stable for up to 3 months and was internalized by HaCaT and SW480 cells to serve as a substrate for the generation of protoporphyrin IX [64]. In a study by Corrêa et al. (2024), PMVEMA-gelatin sponges loaded with ellagic acid demonstrated antibacterial and bioadhesive properties for wound bandaging [78]. In another study by Varshosaz et al. (2017), PMVEMA/PLGA blended nanofibers loaded with montelukast showed hemostatic effects and supported cell proliferation without cytotoxicity [66]. In vaginal health, 3D-printed devices containing a PMVEMA layer showed a 5-fold increase in mucoadhesion, enhancing the residence time of metronidazole for treating bacterial vaginosis [79].

The ionizable carboxyl groups in PMVEMA (pKa~5.3) make it an ideal candidate for pH-responsive smart carriers. Fluorescent nanogels derived from PMVEMA-Es swell at physiological pH and can retain or release drugs based on environmental acidity [80]. In another study by Yang et al. (2024), macrophage membrane-coated PMVEMA nanoparticles were designed to respond to the low pH of the tumor microenvironment, selectively releasing doxorubicin within oral squamous cell carcinoma cells [81].

**Table 3:** Studies using PMVEMA for drug loading purposes.

Type of Drug	Drug	Biomaterial	Effect	Ref
Analgesic	Capsaicin	PMVEMA-Es NFs	100% EE; conservation and improvement of the capacity to activate the TRPV1 channel.	[82]
	Methyl salicylate			
	Salicylic acid			
Antiangiogenic	Bevacizumab	HSA NPs coated with PMVEMA-Es425	99.5% EE; 30% released in 24 h.	[60]
	Suramin		85.3% EE; 80% released in 8 h.	
Antidiabetics	Glibenclamide	Cyclodextrins-modified PMVEMA NPs	60% EE; hypolipidemic effect through activation of insulin signaling pathway in *Caenorhabditis elegans.*	[46]
	Insulin	Zein NPs coated with PMVEMA-PEG	87% EE; improved diffusion in intestinal mucus; reduction in glucose content and fat accumulated in *C. elegans*.	[76]
Anti-inflammatory	L-menthol	PMVEMA (119 and 139) NFs	92 (139) and 68% EE; TRPM8 activation levels equivalent to free L-menthol.	[83]
Antimicrobial	Metronidazole	3D printed device using PCL and PMVEMA layers	Mucoadhesion increased 5× after incorporation of PMVEMA. Inhibition of *Gardnerella vaginalis* in 5% metronidazole 3D-printed discs.	[79]
	Rifampicin	PMVEMA119-ethyl cellulose NPs	%EE > 82%; targeted lung delivery through Peyer’s Patch uptake.	[49]
Antimigraine	Zolmitripan	Lipomer-PMVEMA119 NPs	86.2% EE; transdermal application showed high bioavailability and similar T_max_ to I.V. route, with higher brain/blood ratio.	[84]
Antineoplastic	Cabacitaxel	PMVEMA-TPGS copolymer in PLGA/lipid NPs	92.1% EE; strong bioadhesion that improved intestinal permeability and oral bioavailability.	[59]
	Carmustine	PMVEMA-Es NFs	80% EE; increase in the S-phase population in glioblastoma cell lines, suggesting cytostatic effect.	[77]
	Docetaxel	PMVEMA-PEG NPs	60% EE; high plasma levels after oral administration. Less toxicity compared to free I.V. administered drug to mice.	[50]
	Doxorubicin	Biotinylated chitosan/PMVEMA NPs	71% EE; reduction in IC_50_ in HepG2 cells compared with free drug.	[85]
		Polyethylene sebacate/PMVEMA NPs with ASGPR ligands.	High liver accumulation (hepatocytes). ASGPR-mediated uptake in HepG2. Sustained reduction in tumor volume.	[86]
		Macrophage membrane-encapsulated with PMVEMA-phenylboronic acid-doxorubicin NPs	54.6% EE; OSCC inhibition, controlled release in low-pH tumor, prolonged circulation time, and diminished immune clearance. Stronger antitumor activity than free doxorubicin.	[81]
		PMVEMA-Ac NFs	100% EE; increase in the SubG1 population in glioblastoma cell lines, indicative of cell death.	[77]
Vasodilator	Vardenafil hydrochloride	Lipid/PMVEMA lipomers	63% EE; delayed time to achieve maximum concentration and extended mean residence time.	[53]

Abbreviations: ASGPR: asialoglycoprotein receptor; EE: encapsulation efficiency; HSA: human serum albumin; I.V.: intravenous; NFs: nanofibers; NPs: nanoparticles; OSCC: Oral Squamous Cell Carcinoma; PCL: polycaprolactone; PEG: polyethylene glycol; PMVEMA: poly(methyl vinyl ether-alt-maleic anhydride); TPGS: D-α-Tocopheryl polyethylene glycol succinate; TRPM8: Transient Receptor Potential Melastatin 8 ion channel; TRPV1: Transient Receptor Potential Vanilloid 1 ion channel.

### 6.3. Odontology

PMVEMA plays a key role in dentistry because of its bioadhesive properties and its ability to form protective films.

One of its most common uses in toothpaste and mouthwashes is to enhance the retention and efficacy of triclosan. PMVEMA acts as a carrier agent that increases the substantivity of triclosan, allowing this antibacterial agent to adhere to oral surfaces and remain active for periods of up to 12 h [87]. This combination is highly effective in reducing bacterial plaque and gingivitis [88].

Furthermore, due to its strong adhesive properties, some PMVEMA derivatives, such as calcium/sodium PMVEMA copolymer, have been used as ingredients in denture adhesive formulations [89]. In a study by Bezerra et al. (2019), PMVEMA was also investigated as an anti-erosive agent due to its excellent film-forming properties [90]. The polymer significantly reduced surface loss in both enamel and dentine under erosive conditions, showing superior protective effects compared with other polymers, such as propylene glycol alginate, poly(vinylpyrrolidone) and carboxymethylcellulose. Moreover, a two-year clinical trial evaluated a dentifrice containing 0.3% triclosan and 2% PMVEMA during the maintenance phase following peri-implantitis treatment [91]. The formulation promoted peri-implant clinical attachment stability, reduced probing depth and bleeding on probing, and prevented progressive bone loss around treated implants, demonstrating superior outcomes compared with conventional fluoride toothpaste. In addition, it significantly decreased the levels of pathogenic red-complex bacteria, including *Porphyromonas gingivalis*, *Treponema denticola* and *Tannerella forsythia*. Along the same lines, PMVEMA demonstrated significant anti-adherent activity against oral bacteria by reducing the attachment of *Streptococcus sanguis* and mixed oral microflora to hydroxyapatite surfaces, which simulate tooth enamel [92].

### 6.4. Dermatology

PMVEMA has also been used in the development of transdermal delivery systems, particularly MN patches and adhesive patches, serving as a structural support and providing mechanical strength in the manufacture of MNs [93]. This allows them to be strong enough to penetrate the stratum corneum without breaking. Formulations with higher concentrations of PMVEMA typically exhibit superior mechanical stability under compressive forces [93].

Several studies have highlighted the advantages of PMVEMA-based MN systems. Saepang et al. (2021) reported that PMVEMA/PVA formulations produced mechanically robust MNs with excellent skin insertion capability and efficient formation of skin microchannels [94]. The MN systems markedly enhanced transdermal drug permeation compared with intact skin, enabling sustained and controlled drug delivery of pramipexole dihydrochloride. Similarly, Sabri et al. (2021) developed hydrogel-forming MNs based on PEG/PMVEMA and sorbitol for drug monitoring applications [95]. These systems exhibited excellent mechanical properties together with efficient extraction capabilities, highlighting their potential as minimally invasive platforms for therapeutic monitoring.

Comparative studies have further demonstrated the advantages of PMVEMA over other polymeric matrices commonly used in MN fabrication. PMVEMA-based MNs generally exhibit deeper skin penetration and more sustained drug release than PVP-based systems [96]. While PVP formulations typically show a rapid release plateau, PMVEMA matrices allow a more prolonged and linear drug delivery profile. For example, PMVEMA-based patches loaded with donepezil delivered nearly three times more drug after 24 h (34.63%) than PVP-based patches (13.11%) [96]. A similar effect was observed with zanamivir-loaded systems, where PMVEMA/PEG hydrogels achieved the highest permeation rates, reaching approximately 22%, substantially exceeding the release efficiency of PVP/PVA-based hydrogels and formulations containing additives that restrict swelling [97].

Beyond MN systems, PMVEMA has also been incorporated into adhesive transdermal patches. PMVEMA/poly(N-vinylpyrrolidone-co-acrylic acid) drug-in-adhesive patches containing ketoprofen showed significantly improved skin adhesion compared with commercially available patches [98]. In addition, PMVEMA-based materials have demonstrated promising properties for wound care applications. PVA/PMVEMA cryogels exhibited excellent swelling capacity, favourable mechanical strength, and strong adhesion to the skin, together with a soft consistency and high moisture content [37].

## 7. Industrial Interest

The translational potential of PMVEMA is clear due to the combination of regulatory acceptance for some applications, its commercial use, and continued industrial innovation. As discussed previously, PMVEMA and several of its derivatives are associated with recognized or authorized uses in a variety of applications involving direct or indirect human exposure, including food-contact materials, denture adhesives, and cosmetic formulations. Importantly, the polymer has accumulated decades of practical use in consumer products, providing a substantial body of safety and performance data that can facilitate the development of new biomedical applications.

The widespread commercial use of PMVEMA is particularly evident in the cosmetic industry. Current cosmetic-use inventories indicate that PMVEMA copolymer and related esters are incorporated into numerous formulations, including hair sprays, styling products, binders, film-forming agents, viscosity modifiers, and emulsion stabilizers [6]. Depending on the derivative and formulation, concentrations may range from trace amounts to as high as 30%. Furthermore, these ingredients are not subject to specific restrictions in either the European Union or Japan, reflecting their established safety profile for cosmetic use. Beyond cosmetics, PMVEMA has also been employed in a variety of industrial applications, including adhesives, coatings, detergents, paper and textile processing, and is being investigated for ophthalmic drug-delivery systems [99]. PMVEMA and some of its derivatives are listed in the FDA Inactive Ingredient Database for use in topical formulations at concentrations of up to 30% [100].

**Figure 6:**
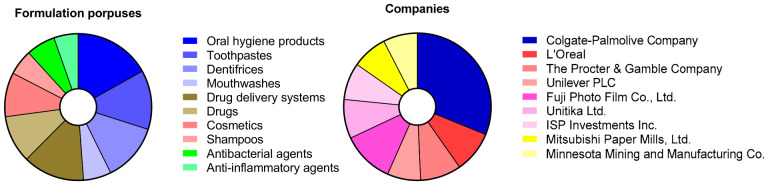
Distribution of patents found in CAS SciFinder by the top ten formulation purposes and assignee companies.

The extensive history of commercialization is mirrored by a remarkable patent landscape. A search of the CAS SciFinder database conducted on 24 June 2026 using the commercial name of PMVEMA yielded more than 3500 patent documents, of which approximately 1000 were active, demonstrating sustained technological and commercial interest. Notably, the distribution of patents closely reflects the sectors in which PMVEMA has achieved the greatest market penetration, as shown in Figure 6. Oral-care applications dominate the portfolio, accounting for approximately 17% of patents related to oral hygiene products, 13% to toothpastes, 13% to dentifrices, and 6% to mouthwashes. This predominance is consistent with the well-established bioadhesive, film-forming, and antibacterial properties of PMVEMA, which have enabled its successful incorporation into numerous commercial dental products. Another well-represented application field is drug delivery, with approximately 14% of patents related to drug delivery systems and 10% to drugs. Finally, cosmetics account for 10% of the patents.

Patent ownership is concentrated among major multinational companies, with Colgate-Palmolive holding approximately 31% of the portfolio, followed by L’Oréal and Procter & Gamble with approximately 9% each. The continued generation of intellectual property by these companies over several decades indicates that PMVEMA remains an active platform for innovation rather than a mature technology that has reached commercial saturation. The persistence of patent activity also suggests that the polymer continues to offer opportunities for product differentiation and technological advancement in highly competitive consumer healthcare markets.

Interestingly, despite the extensive scientific literature describing PMVEMA-based nanoparticles, vaccine adjuvants, hydrogels, MNs, and tissue-engineering scaffolds, relatively few advanced biomedical products based on these technologies have reached the market. Commercial applications remain concentrated in oral care and cosmetics, where regulatory pathways are generally more straightforward and the performance of the polymer is already well established. This discrepancy highlights a translational gap between academic research and commercial implementation that may be related to the complexity of advanced formulations, manufacturing challenges, regulatory requirements for novel drug-delivery systems, and the still incomplete understanding of the long-term biodegradation, biodistribution, and environmental fate of PMVEMA and its degradation products.

## 8. Conclusions and Future Perspectives

PMVEMA and its derivatives have emerged as highly versatile polymeric platforms with broad applicability in biomedicine and pharmaceutical technology. The extensive literature published over several decades demonstrates sustained scientific interest in this polymer and highlights its continuous technological evolution. The unique reactivity of its anhydride groups enables straightforward functionalization and crosslinking, facilitating the development of multifunctional biomaterials. In addition, PMVEMA exhibits intrinsic bioadhesive, mucoadhesive, and, in some formulations, antimicrobial properties that distinguish it from many conventional synthetic polymers. Nevertheless, several limitations remain, including restricted loading of highly hydrophobic compounds, excessive mucoadhesion in certain delivery applications, and relatively rapid hydrolytic degradation. Importantly, many of these challenges can be mitigated through formulation strategies such as polymer functionalization and chemical or ionic crosslinking.

These properties have enabled the successful application of PMVEMA-based systems across a wide range of biomedical fields. In drug delivery, PMVEMA has been employed to improve bioavailability and controlled release through oral, dermal, transmucosal, and transdermal administration routes. In dentistry, it has found applications in denture adhesives and anti-biofilm technologies. PMVEMA-based materials have also shown promise in dermatology, vaccine delivery, and immunotherapy. Notably, some PMVEMA derivatives have demonstrated intrinsic immunomodulatory activity, acting as Th1-oriented adjuvants through activation of Toll-like receptors and the complement system, further expanding their therapeutic potential.

The translational potential of PMVEMA is supported by its long history of use in products intended for human exposure and by the significant industrial interest surrounding the polymer. Several PMVEMA-based materials and formulations have received regulatory acceptance, or are associated with recognized uses, for specific applications, including food-contact materials, denture adhesives, and cosmetic products. Furthermore, the extensive patent activity associated with PMVEMA technologies, particularly in oral-care products, reflects the continued commercial interest of major industrial stakeholders. However, despite the large number of scientific publications and patents, relatively few advanced PMVEMA-based biomedical products have reached clinical practice, highlighting the challenges associated with regulatory approval, manufacturing scalability, and product development in this field.

**Figure 7:**
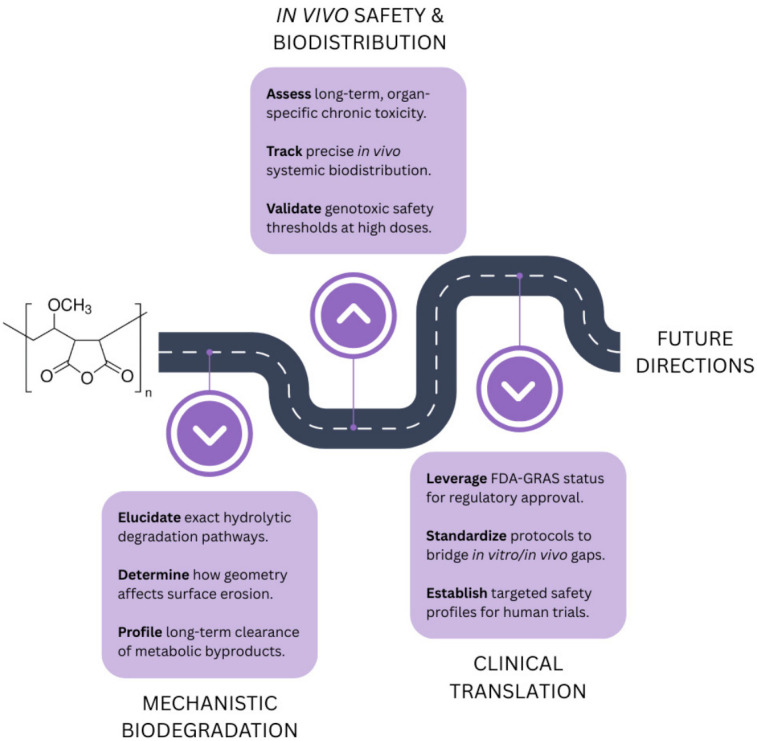
Future directions for the development of PMVEMA-based systems.

Future research should focus on addressing the remaining knowledge gaps that limit the broader translation of PMVEMA-based technologies. In particular, a deeper understanding of biodegradation mechanisms and in vivo safety, biodistribution, and long-term fate remains necessary, together with studies addressing manufacturing scalability, regulatory requirements, and clinical translation, as summarized in Figure 7.

## Figures and Tables

**Table 1:** Summary of the main properties of PMVEMA for use in biomedical applications.

Properties	Brief Description	Ref
Biocompability	Low cytotoxicity, minimal effects on cell viability and membrane integrity, absence of significant genotoxicity or mutagenicity, and limited systemic absorption.	[33]
Bioadhesion	Strong adhesion to mucosal surfaces through hydrogen bonding between carboxylic acid groups and mucins.	[34,35]
Degradation	Hydrolytic degradation through cleavage of anhydride bonds, generating dicarboxylic acids. Degradation generally occurs through surface erosion.	[36]
Antimicrobial	Activity is associated with the acidic microenvironment generated by hydrolyzed carboxylic groups and with inhibition of bacterial adhesion and biofilm formation.	[37,38]

**Table 2:** Limitations of PMVEMA and proposed strategies to overcome them, as documented in the literature.

Limitation	Solution Proposed	Ref
- Low loading of highly lipophilic drugs	- Co-encapsulation with cyclodextrins. - Preparation of mPEG–drug conjugates.	[46,47,48,49]
- Excessive mucoadhesion can reduce the permeability of mucosal barriers	- PEGylation; coating with galactose-containing polysaccharides. - Incorporation of hydrophobic excipients	[50,51]
- Fast drug release	- Chemical crosslinking with diamines. - Ionic crosslinking with divalent cations.	[48,52,53]

## Data Availability

The original contributions presented in this study are included in the article. Further inquiries can be directed to the corresponding author.

## Abbreviations

The following abbreviations are used in this manuscript:

5-ALA	5-aminolevulinic acid
APC	antigen-presenting cells
BCNU	carmustine
BSA	bovine serum albumin
DC	dendritic cells
DOX	doxorubicin
EDC	1-ethyl-3-(3-dimethylaminopropyl)carbodiimide
FDA	Food and Drug Administration
GRAS	Generally Recognized as Safe
HA	hyaluronic acid
HP-β-CD	hydroxypropyl-β-cyclodextrin
IL	interleukin
MN	microneedle
NALT	nasopharynx-associated lymphoid tissue
NHS	N-hydroxysuccinimide
PEG	polyethylene glycol
PLGA	poly(lactic-co-glycolic acid)
PMVEMA	poly(methyl vinyl ether-*alt*-maleic anhydride)
PMVEMA-Ac	poly(methyl vinyl ether-*alt*-maleic acid)
PMVEMA-Es	poly(methyl vinyl ether-*alt*-maleic acid monoethyl ester)
PVA	poly vinyl alcohol
PVP	poly(vinylpyrrolidone)
TLR	toll-like receptor
TNF	tumor necrosis factor
TPGS	D-α-Tocopheryl polyethylene glycol succinate
SALT	skin-associated lymphoid tissue
WGA	wheat germ agglutinin
